# Supplementary material for: Exploring FeLV-Gag-Based VLPs as a New Vaccine Platform—Analysis of Production and Immunogenicity
Source: Int J Mol Sci. 2023 May 19;24(10):9025. doi: 10.3390/ijms24109025 (PMC10219511; doi:10.3390/ijms24109025)
Supplement: Supplementary file 1 [file ijms-24-09025-s001.zip › ijms-2373577-supplementary.pdf]

International Journal of
Molecular Sciences

MDPI

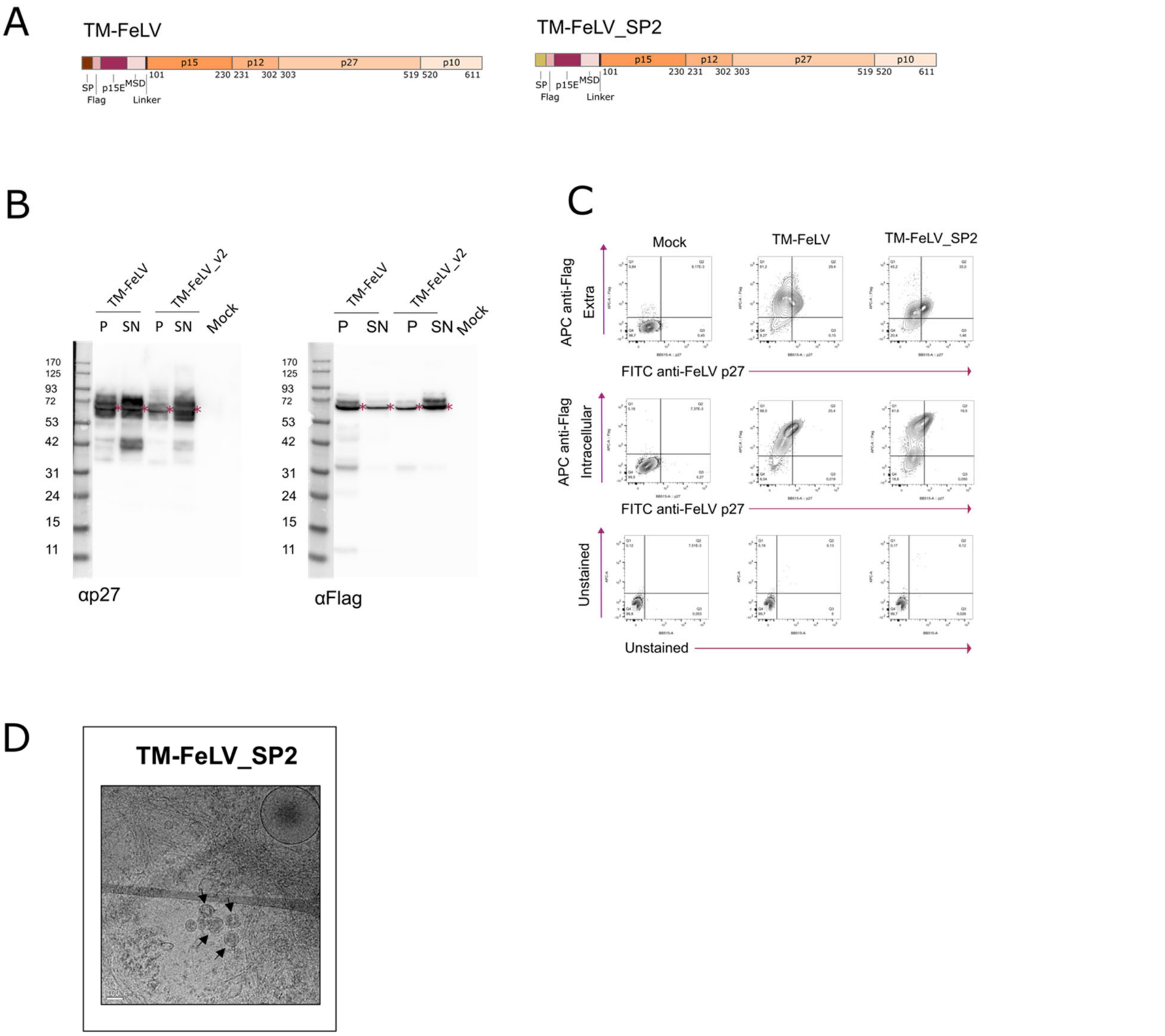

Figure S1. Comparison between two different signal peptides. (**A**) Schematic representation of the fusion proteins TM-based VLPs with two different signal peptides (SP). TM-FeLV presents GMCSF SP (brown), and the TM-FeLV_SP2 fusion protein presents the immunoglobulin H5 SP (gold). (**B**) Western blot was developed with anti-p27 or anti-Flag antibodies to analyze the expression of TM-based fusion proteins transiently transfected in Expi293F cells. Asterisks indicate the expected molecular weight of fusion proteins. (**C**) Representative flow cytometry panels for expression of the fusion proteins. Upper panel: identification of Flag Tag on the surface of the cells and intracellular Gag. Middle panel: identification of intracellular Flag tag and Gag. Lower panel: unstained as a control. (**D**) Cryo-EM images of the extracted and purified VLPs from the cell pellet are shown.

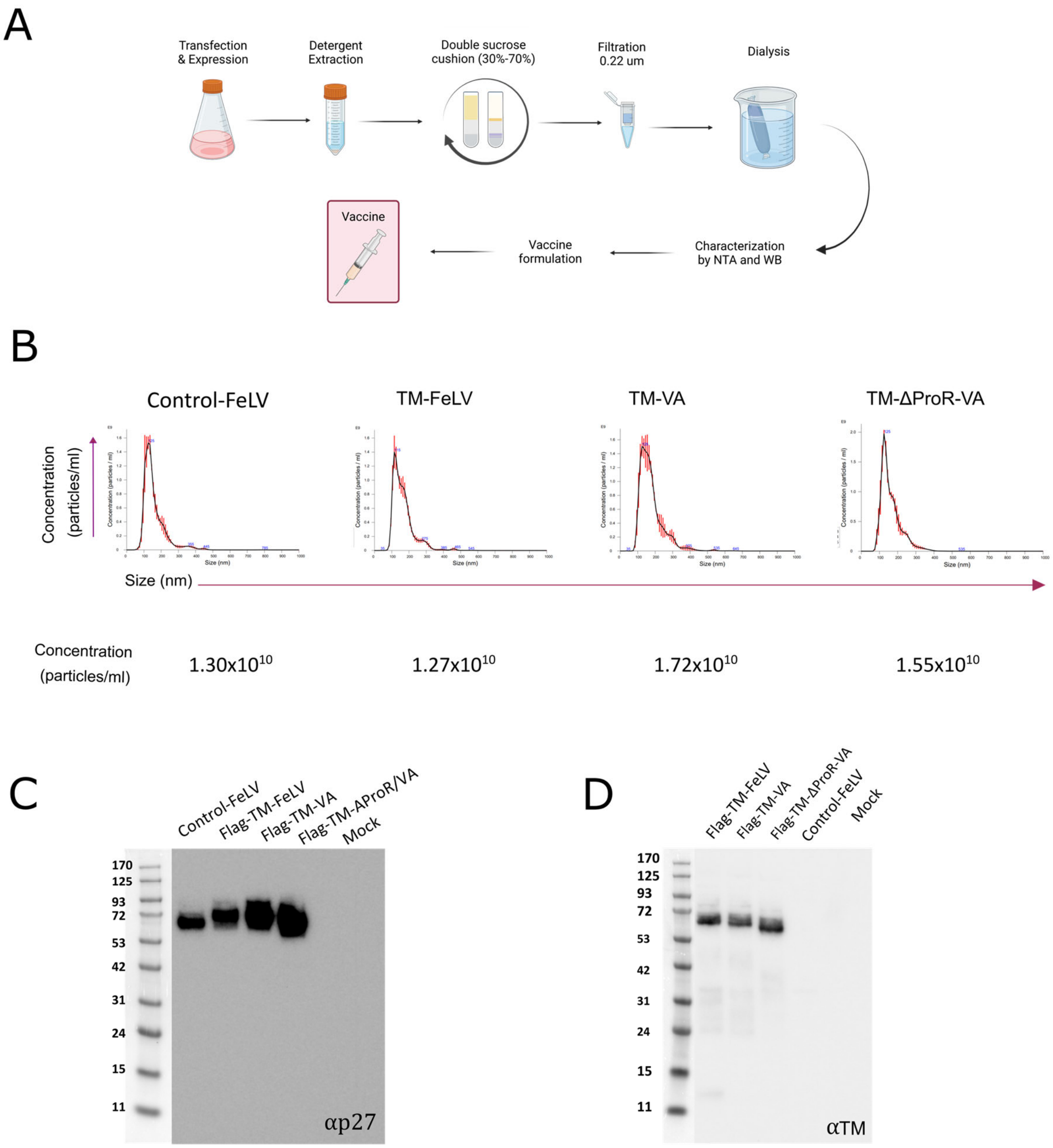

Figure S2. Quantification and analysis of VLPs by NTA, and vaccine preparation. (**A**) Graphical scheme of the experimental procedure for the preparation of VLP vaccines: production, extraction, purification, quantification, and vaccine formulation. Created with BioRender.com. (**B**) Estimation of size and concentration of the final preparation performed by NTA. Western blots developed with anti-p27 mouse monoclonal antibodies (**C**) and anti-TM rabbit polyclonal antibodies (**D**) to analyze the expression of the vaccine preparation before formulation.

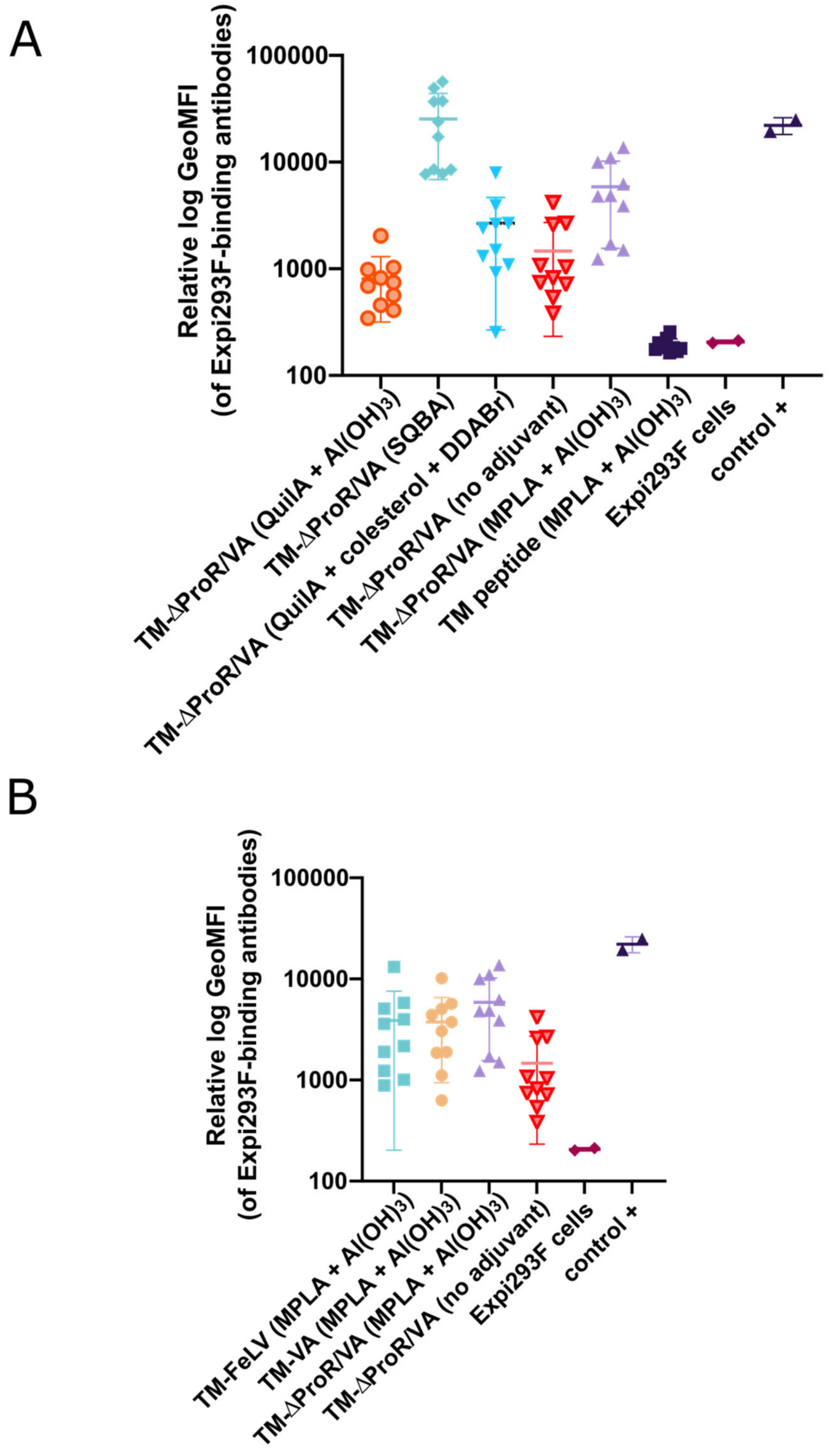

Figure S3. Humoral immune response against Expi293F host cell proteins. Levels of IgG against Expi293F host cell proteins analyzed by flow cytometry. Data are presented as geometrical mean fluorescence intensity (Geo-MFI) ± SD. (**A**) Response of immunization with TM-ΔProR-VA plus different adjuvants. (**B**) Response of immunization with the selected fusion proteins adjuvanted with MPLA + $Al(OH)_3$.

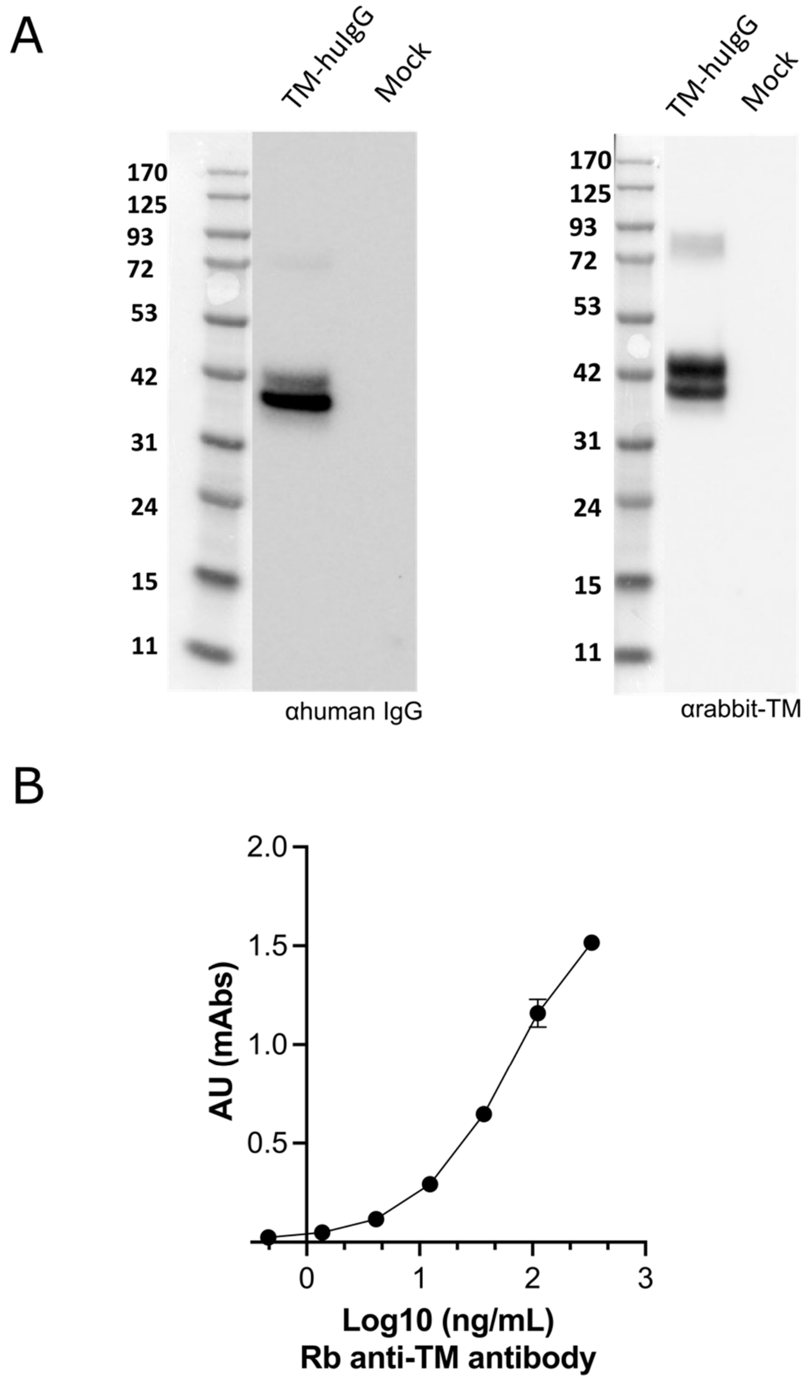

Figure S4. Western Blot of TM-huIgG. (**A**) Western blot developed with anti-human IgG or rabbit anti-TM antibodies to analyze the expression of the TM-huIgG fusion protein transiently transfected in Expi293F cells. (**B**) Recognition of the TM-huIgG by anti-TM rabbit polyclonal antibodies measured by ELISA.
